# The Ethical Schism in New York

**Published:** 1900-09

**Authors:** 


### THE ETHICAL SCHISM IN NEW YORK.

THE journals have contained much of late regarding the relationship of the two state medical bodies that claim coequal powers under the law, much of which is misleading and some of which is absolutely devoid of truth. The fact is that very few outside of this state know that the elder organisation—The Medical Society of the State of New York—has been the recognised professional and official society since 1806, and that the younger body—The New York State Medical Association—was organised in 1884.

Last winter the latter obtained a charter from the legislature and has given out that it is equal in legal power with the parent society. This question of legal rights and power is now being tested in the courts, which alone can decide disputed law points, and until the decision is finally handed down from the highest tribunal, opinions in the premises will be valueless, hence had better be reserved.

As bearing on the question of ethical differences that separate the two state organisations, our esteemed contemporary, the *New York Medical Journal*, in its issue of August 18, 1900, has spoken most wisely in its editorial columns. We esteem it a pleasure to reprint the article in question, which is as follows:

THE AMERICAN MEDICAL ASSOCIATION AND THE MEDICAL SOCIETY OF THE STATE OF NEW YORK.

Except for the ebullition which occurred at the Denver meeting of the American Medical Association, a discreet silence has been observed for several years concerning the forfeited affiliation of the Medical Society of the State of New York with the national organisation. There has been a general feeling that time alone would heal the breach that is more apparent than real. If anybody was qualified to break that silence, it was Dr. Jacobi, and he has ventured to do so in an address delivered before the International Medical Congress in Paris. Dr. Jacobi's statement of the case is clear and candid, and his long and spotless career will give weight to his words, albeit they savor of the pathetic.

Evidence has for some time been accumulating that the profession is weary of the awkwardness which is sometimes the unavoidable outcome of the position in which the New York Society placed itself some years ago. We believe that the American Medical Association is rapidly approaching the attitude of being willing to modify its code of ethics to suit changed conditions, and certainly there have been vast changes since the time when the present code was adopted. We believe, too, that the Medical Society of the State of New York, while unable to take the initiative in the movement for reconciliation, is prepared to welcome any conciliatory measures that the central organisation may feel like taking. We earnestly hope that we are not mistaken in our interpretation of the signs on which we found our auspicious forecast, and we know, that that hope and that interpretation are shared by a great number of the prominent members of the profession throughout the country. We feel sure, also, that the physicians of the State of New York have no such false pride as would prompt them to look askance upon the olive branch. The present situation is anomalous, it is monstrous; it may be righted speedily and thoroughly if men who are friends in private life can bring themselves to mutual concession in corporate action. Several months are yet to elapse before the St. Paul meeting is held. Let us hope that that meeting will put an end to the estrangement between the American Medical Association and the Medical Society of the State of New York.

The *Journal*, as will be observed, makes the address of Dr. Abraham Jacobi, at the 13th International Medical Congress, at Paris, the basis of its remarks. Dr. Jacobi is familiar with the whole question, was a prominent actor in the scenes preceding the division in the old society and the formation of the new association, and knew whereof he spoke. Moreover, his high standing throughout the world, entitles him to a considerate hearing. Therefore we reproduce a portion of his address, taken also from the issue of the *Journal* previously referred to. We invite a careful reading of it, which is as follows:

MEDICAL ETHICS IN THE UNITED STATES.

The ethical conscience of the physicians of the United States is exhibited in a great many ways. It is considered unethical for a doctor to own a drug store or a part of it; ever to take a patent on any invention of his own; ever to recommend over his name a patented instrument, a proprietary food, or medicine, or mineral water. That is illustrated by what happened to the famous Morton after his successful demonstration of the anesthetic effect of ether in the Massachusetts Hospital. After having extracted a tooth on September 30, 1846, without giving the patient any pain, he applied immediately for patent rights and sold individual office rights. Moreover, he kept his composition, which he called 'letheon,' a secret. It was then that the profession turned against him and forced him to admit that his 'letheon' was sulphuric ether disguised by some aromatic oil. A medical man in America is forbidden ever to advertise his name, or office hours, or his specialty in a newspaper or lay magazine, ever to announce his specialty, if any he have, on his cards. If reports of cases or operations of same physician appear repeatedly in the public press, it is taken for granted that it has not been without his knowledge, consent or prompting. All of these things impair a medical man's professional standing; they render him an improper candidate if ever he applies for membership in a medical society, or make him, if he be a member, the subject of discipline.

Many of the rules accepted by American medical men are contained in a book compiled by an Englishman, Percival, in 1807. It was received as the code of ethics of the American Medical Association in 1847, and is still obeyed by those who know the book or who do not know it, or even by those who never know of its existence. One of the regulations of the code forbids consultations with homeopaths.

It is hardly necessary to say to those of you for whom the practice of medicine is not only diagnosis and autopsy, but the treatment and cure of the patient in whose behalf a consultation is to be held, that when medical treatment is in question you cannot agree with a homeopath who is a Hahnemannian; and you do not want to meet a homeopath who, because the name is still fashionable and for a portion of the misinformed public the subject of an almost religious fanaticism, employs that title for meretricious purposes. Still there are cases in which it would be inhuman to refuse a consultation. Not only were such consultations held from olden times, but even in large cities exceptions to the rule have always been frequent, indeed too frequent in my opinion. Moreover, whoever is acquainted with smaller cities and villages, where a homeopath is the only rival or companion of the regular physician, knows that for either it would be suicidal to refuse a consultation. Only lately one of the medical men most widely know for his wisdom, in the American Medical Association and the profession at large, Dr. S. C. Busey, of Washington, proclaimed that the rule forbidding such consultations should be so modified or explained as to permit them in cases of emergency. This is exactly what the Medical Society of the State of New York made its official policy in 1882. We considered it more honest to admit by law what was constantly being done, and to decree precisely that for the reasons of humanity and in an urgent case a consultation should not be refused. For this sin we, the body of the Medical Society of New York, were expelled from the American Medical Association.

